# An Invited Reply to: A Comment on: An Eocene army ant (2022) by Sosiak CE *et al*.

**DOI:** 10.1098/rsbl.2023.0140

**Published:** 2023-04-26

**Authors:** Christine E. Sosiak, Marek L. Borowiec, Phillip Barden

**Affiliations:** ^1^ Federated Department of Biological Sciences, New Jersey Institute of Technology, Newark 07102-1982, USA; ^2^ Department of Agricultural Biology and C. P. Gillette Museum of Arthropod Diversity, Colorado State University, Fort Collins, CO 80523, USA; ^3^ Division of Invertebrate Zoology, American Museum of Natural History, New York, NY 10024, USA

We first establish that we agree with a central concern of Dubovikoff & Zharkov [1] regarding the provenance of the army ant specimen we reported [2]. Indeed, the collection the authors note, housed within the Zoological Institute of the Russian Academy of Sciences (St Petersburg), is one of two collections we were made aware of shortly after the publication of our article. Although not mentioned in their reply, we were informed by one of the authors that the copal within this collection was initially dated as Baltic amber in the 1920s. This collection date was concerning as the specimen we reported, housed at the Museum of Comparative Zoology and labelled as Baltic amber, was included in a museum ledger from the 1930s. News of this historical collection at the Russian Academy of Sciences, as well as another in Germany from the same timeframe, prompted us to investigate the provenance of our reported specimen via Fourier-transform infrared (FTIR) spectroscopy. Our FTIR analyses indicated that the specimen we reported did not possess a unique spectroscopic characteristic of Baltic amber and was most likely subfossil resin (copal); these results constituted the basis of our retraction [3].

There is no doubt that the specimen is not Baltic amber; however, there are three elements that Dubovikoff & Zharkov raise that we feel are inaccurate and require a response in the literature [1]. These issues relate to the identification of copal specimens, CT-scan segmentation and repeatability, and the nature of taxonomic assignment.

## Baltic amber and copal cannot always be discerned by eye or with basic physical tests

Dubovikoff & Zharkov suggest that the specimen could have been identified as copal through visual assessment or what the authors refer to as a ‘simple test’ involving polishing or exposure to ethanol. The authors note that the cracks visible on the surface of the specimen (also known as crazing) suggest that it is a young resin such as copal. It is true that copal can craze rapidly relative to amber; however, authentic Baltic amber can exhibit such crazing, particularly historic specimens exposed to light or humidity fluctuations [4,5]. The authors note that the copal specimens they have studied melt during polishing and/or exposure to ethanol. While we did not undertake polishing or an ethanol trial, the specimen we reported was affixed to a microscope slide. The identity of the slide mountant is not known to us; however, it was treated throughout with some kind of tacky material. Historically, Baltic amber researchers used Canada balsam or other resins to affix amber to slides [5,6]. Canada balsam and other resin mountants are soluble in ethanol and would melt during polishing, rendering these indicators unreliable unless one were to trim deep into the specimen. We noted in the initial paper that no preparation was undertaken to avoid further damage due to crazing or chemical reactions to previously applied polymers. While the provenance test suggested may be useful in some cases, it is not a definitive approach. If an amber piece was previously treated with an unknown substance, using solely the criteria suggested by Dubovikoff & Zharkov may lead to instances of false negatives: incorrect identification of copal where genuine Baltic amber is present. For this reason, we turned to FTIR spectroscopy to definitively assess the provenance of the specimen after learning of other mislabelled copal material.

## CT-scan segmentation includes a trade-off between precision and accuracy

**Figure 1.**
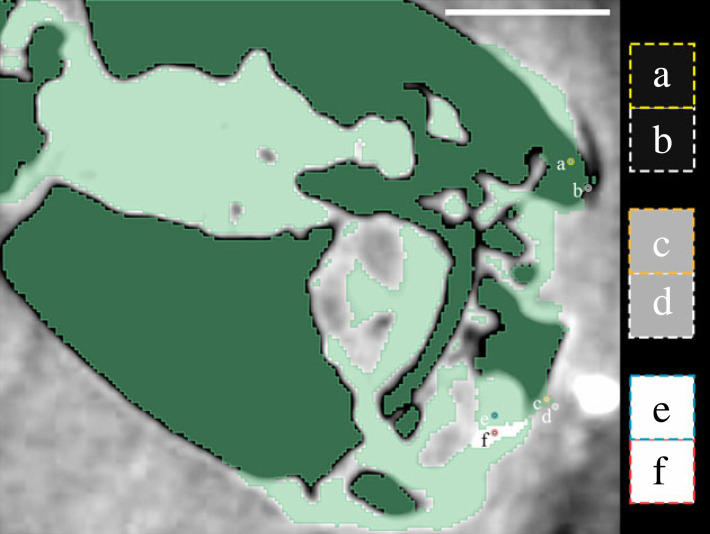
Z-stack data from the CT scan of PALE-8463. (Left) Cross-section of the head at the anterior margin; segmented elements from the three-dimensional reconstruction of Dubovikoff & Zharkov [1] in green. (Right) Squares infilled with grey-scale values demonstrating examples of nearby pixels with identical or near identical grey values that were both included and excluded in the manual segmentation process. Labels on squares denote associated pixels. Scale = 0.25 mm.

In CT-scan reconstruction, X-ray attenuation and absorption is denoted solely as relative grey values of pixels in two-dimensional images. These two-dimensional images comprise a series of slices termed a Z-stack. Any three-dimensional representation of CT-scan data is the result of selectively rendering certain pixels within this stack. Because it is not possible to resolve differences between pixel grey values at critical morphological features in our scan, we chose to present a three-dimensional representation of a clearly differentiated selection of pixels within our Z-stack data: the internal void space within the specimen. Amber and copal-entombed insects are frequently preserved as thin shells of cuticle with air trapped inside. X-ray transmission is starkly different between these void space regions compared to insect cuticle and resin matrix, which share similar X-ray attenuation properties. This stark difference allows for a clear delineation that allows for a conservative and, importantly, repeatable segmentation process. Using the published Z-stack slices from Sosiak *et al*. [2], Dubovikoff & Zharkov generated what they term a ‘complete’ or ‘full model reflecting the true structure of the described specimen’. There is no question that they presented more features in their final segmentation; however, the authors indicate that this was done by manually segmenting the scan (i.e. drawing one's interpretations of what is and is not valid inclusion structure). While this approach can remove artefacts, it also introduces subjective interpretations into the segmentation process and reduces repeatability [7]. Three-dimensional reconstructions of CT-scan data are hypotheses and include inherent assumptions. A method of manual segmentation may be appropriate when a large series of putative conspecific specimens can guide interpretations, but such a segmentation can contain inconsistent and subjective criteria for what is and is not included in a final model (figure 1). With a single specimen of putatively unique geological origin, we presented a model with the least number of assumptions that was fundamentally repeatable. We did rely on void space to delineate some regions (particularly the mandibles and frontal lobe shape) included in our taxonomic diagnosis; however, this was only after we could not resolve these regions via light microscopy or through assessment of grey values within raw Z-stack slices themselves.

## Establishing a conspecific without diagnostic features and name validity

We agree in principle that our reported taxon should be synonymized with *Dorylus* Fabricius, 1793. However, we were recently made aware that, as the initial description was published in an electronic format journal, the names are not considered valid under the International Commission on Zoological Nomenclature (ICZN) unless registered with ZooBank [8]. We did not complete such registration and so the synonymizations proposed by Dubovikoff & Zharkov do not hold authority under the current ICZN Code [8]. Moreover, we do feel that more data and clear diagnostic features are needed to establish that the specimen we reported is conspecific with an extant taxon, particularly as there does not appear to be a clear locality or age estimate associated with the copal specimens reported so far.

## Data Availability

This article has no additional data.
